# Effect of Different Percentage of *Camelina sativa* Cake in Laying Hens Diet: Performance, Welfare, and Eggshell Quality

**DOI:** 10.3390/ani10081396

**Published:** 2020-08-11

**Authors:** Susanna Lolli, Guido Grilli, Lorenzo Ferrari, Giovanna Battelli, Sara Pozzo, Incoronata Galasso, Roberto Russo, Milena Brasca, Remo Reggiani, Valentina Ferrante

**Affiliations:** 1 Department of Environmental Science and Policy, Università degli Studi di Milano, via G. Celoria 2, 20133 Milano, Italy; susanna.lolli@dsm.com (S.L.); lorenzo.ferrari@unimi.it (L.F.); 2 Department of Veterinary Medicine, Università degli Studi di Milano, via dell’Università 6, 26900 Lodi, Italy; guido.grilli@unimi.it; 3 National Research Council, Institute of Sciences of Food Production, via G. Celoria 2, 20133 Milano, Italy; giovanna.battelli@ispa.cnr.it (G.B.); sara.pozzo@ispa.cnr.it (S.P.); milena.brasca@ispa.cnr.it (M.B.); 4 National Research Council, Institute of Agricultural Biology and Biotechnology, via Bassini 15, 20133 Milano, Italy; incoronata.galasso@ibba.cnr.it (I.G.); rob822@libero.it (R.R.); remo.reggiani@ibba.cnr.it (R.R.)

**Keywords:** laying hen, camelina cake, fatty acids, antinutritional compounds, shell stiffness, volatile organic compounds

## Abstract

**Simple Summary:**

Nowadays, it is of primary importance to find alternative and sustainable protein sources for animal feeding, taking into account environmental sustainability and animal welfare and production. *Camelina sativa*, as an alternative source of protein in animal feeding, seems to be a good candidate, but its use is limited by the presence of antinutritional compounds. In this study, a camelina breeding line with a low level of glucosinolates was tested for 31 weeks, in order to verify if the inclusion of up to 20% of camelina cake, in the diet of laying hens, could have an adverse effect on the production performance, eggshell quality, and animal welfare and health. Results demonstrated that the performance was maintained, as well as health and welfare, while eggshell quality slightly improved when hens got older.

**Abstract:**

Although camelina [*Camelina sativa* (L.) Crantz] is a good source of protein, antioxidants, and polyunsaturated fatty acids, its antinutritional compounds limit its use in animal feeding. The aim of this study was to verify the effect of feeding laying hens with up to 20% of camelina cake from a breeding line containing a low level of glucosinolates on performance, welfare, and eggshell quality. Two hundred and forty Hy-Line^®^ hens from 18 to 51 weeks of age were divided into three treatments: control (C), camelina cake 10% (CAM10), and camelina cake 20% (CAM20). Egg number was recorded daily, while egg weight, feed consumption, and mortality were recorded weekly. At 24 and 43 weeks of hen age, shell resistance to fracture was measured. Our results demonstrate no detrimental effects for CAM10 and CAM20 diets on feed intake, growth performance, and welfare. No difference in egg production was detected among the diets. The significant (*p* < 0.05) interaction of diet and age factors suggest that the addition of camelina cake, up to 20%, likely protects the eggshell of older hens. Our findings confirm that camelina cake might be an alternative and sustainable protein source for hens.

## 1. Introduction

To overcome the dependence of the European Union (EU) on external protein supply, it is necessary to find new crops or by-products of plant production and agroindustry rich in proteins. The EU imports about 75% of protein-rich raw vegetable materials that it needs to balance livestock feed rations [1].

Growing concerns about the environmental impact of farming systems have increased the interest in finding alternative protein crops or other plants from which protein by-products can be obtained sustainably in Europe. A report by the European Commission (EC) examined many different crops currently available throughout Europe, which could be grown as new resources of renewable raw material for industrial purposes [2]. Among these alternative crops, many of them resulted in attractive candidates for lowering the deficiency in plant oil and protein production. One candidate is *Camelina sativa* (L.) Crantz, or simply camelina, which contains 35–40% of oil and 25% of crude protein in the seeds [3,4].

Camelina is an oilseed crop belonging to the *Brassicaceae* family and it is native to Southeast Europe and Southwest Asia [5]. Camelina can grow in a variety of climatic and soil conditions and it has many agronomic advantages. It can be easily incorporated into crop rotations; it has a short growing season (100–120 days); it tolerates cold weather, drought, and low-fertility soils; it can be grown on marginal farmland, with low water and nutrient requirements [6,7,8]. For all of the above characteristics, camelina seed oil has been widely recognized as a potential alternative source for biodiesel [9,10] and jet-fuel production [11], whereas the seed cake (a by-product generated after the seed oil extraction), rich in proteins (30–40%), a considerable amount of residual oil (10–15%), carbohydrates, and other phytochemicals [12,13], has been suggested as a new potential ingredient in livestock rations [14,15]. In addition, differently from other widely cultivated *Brassicaceae* (e.g., rapeseed) camelina owns a unique oil profile, rich in α-linolenic acid (>35%) and tocopherols (800–1000 ppm) [16,17]; thus, making its seeds highly suitable for a variety of feed, food, or non-food applications [4].

The use of camelina cake as feed for poultry has been widely explored with very different results regarding the effects on animal performances. For example, Aziza et al. (2010) [18] did not find any difference in body weight gain, carcass weight, or feed efficiency when comparing animals fed with camelina with those fed with a maize-soybean based diet, whereas Ryhänen et al. (2007) [19] and Pekel et al. (2009) [20] found an impaired growth between 15 and 37 days of age. The authors explain these differences to the high content of glucosinolates (GSLs) that can affect feed consumption. Less negative effects were found in egg production if the camelina inclusion was maintained below 10% [21].

From the welfare point of view, it is known that one of the main causes of feather pecking and cannibalism is due to dietary problems. For example, high dietary protein levels associated with sub-deficiency in lysine might favor cannibalism during the finishing stage more than low dietary protein levels [22]. There are indications that diets high in insoluble fiber may reduce cannibalism outbreaks in laying hens [23]. The ability of insoluble fiber to exert these effects is related to particle size as fine grinding diminishes its stimulatory influence on the gizzard. Moreover, it is rather well known the positive effects of n-3 fatty acids (FAs) on bone structure. Tarlton et al. (2013) [24] demonstrated that laying hens who received a short-chain n-3 FAs supplemented diet exhibited reduced keel fractures (60% less than a standard diet at 50 weeks of age).

Shell quality is a very important matter for table-eggs market. To prevent product losses during handling and transport, eggshell mechanical properties have to be ensured. Several internal (genotype, age, deposition time) and external (housing, feed, water) factors affect the quality of shell [25,26]. Among these factors, the animal diets are of great importance to give the correct amount of nutritionals for a good egg quality and shell development. Different sources (organic and inorganic) and type of supplementations (essential oils, plants, micro minerals) to laying hens’ diets have been studied in order to directly improve shell quality [27,28], or improve egg quality with possible indirect effects on shell as well [29]. Some authors [27,30] noted more interesting results on shell quality associated to diet supplementation when older hens were considered. With progressing hen age, in fact, shell quality (in terms of weight percentage, thickness, and mechanical properties) diminishes because of reduced calcium availability and shell structure and ultrastructure modifications [25,31].

The aim of this study was to verify the effect on feed intake, growth performance, welfare, and eggshell quality when laying hens are fed for a long period (31 weeks) with up to 20% camelina cake obtained from a breeding line containing low level of GSLs.

## 2. Materials and Methods

The breeding line camelina, F8CALG28, developed at the Institute of Agricultural Biology and Biotechnology of the National Research Council, was used in this work. It was sown and grown in Alagna (PV), Italy in 2017. After harvesting, the seeds were mechanically pressed to extract the oil and the remaining cake was stored in a cold room at 8 °C until the use. Before diets preparation, the chemical composition of the camelina cake: crude protein, crude fat, ash, crude fiber, and neutral detergent fiber (NDF) was determined by an accredited laboratory (Associazione Regionale Allevatori della Lombardia (ARAL), Crema, Italy) (Table 1).

### 2.1. Birds Housing and Feeding

Two hundred and forty beak-trimmed Hy-Line^®^ brown from 18 to 51 weeks of age were housed in six identical pens, divided in three treatments, located at the experimental facilities of CCVZS (Veterinary Clinical and Husbandry Centre, poultry unit), University of Milan, Lodi. The animals were housed at a stocking density of 6.7 hens/pen under the same environmental conditions; the choice to have few repetitions with a quite large number of animals in each pen was due to avoid any change in management and environment of the animals that could bias the results. The floor of the pens was littered with wood shavings. The light schedule was kept constant with 16Light:8Dark and 15 min of dawn/twilight. The room temperature was maintained at 20–23 °C. The pens were equipped with round feeders, drinkers, and nests. Layer feed and water were available ad libitum. All animals were vaccinated for Marek disease, Newcastle disease, infectious bronchitis, and coccidiosis. The analysis of avian influenza and salmonella were performed, as required by the law. The layers were fed from 21 to 51 weeks of age with three different diets containing 0% (control C), 10% (CAM10), and 20% (CAM20) of camelina cake, respectively. All diets were formulated to be isoenergetic and isoproteic (Table 2).

### 2.2. Characterization of Camelina Cake and Diets

#### 2.2.1. Analysis of Antioxidants

Samples were prepared by extracting camelina cake and diets with ethanol using a ratio of 1:10 (*w*/*v*). Antioxidant activity was determined as 2.2-diphenyl-1-picrylhydrazyl (DPPH) radical scavenging activity according to methodology described by Brand-Williams et al. (1995) [32]. After 100 min of reaction, the reaction mixture was transferred to 96-well transparent plates and absorbance read at 517 nm. DPPH* (scavenging activity %) was calculated as (1 − Abs_sample_/Abs_control_) × 100, where Abs_control_ is ethanol instead of the sample. The total phenolic content (TPC), flavonoids and tannins were extracted and assayed in all samples as previously described by Russo and Reggiani (2018) [33]. Tocopherol isomers were analyzed in High-Performance Liquid Chromatography (HPLC) by direct injection of oil [34]. Samples were loaded into a Kinetex 5μm EVO C18 100A (Phenomenex) column (100 × 4.6 mm) and eluted with 0.8 mL/min MeOH 93%. Tocopherols were quantitated at 290 nm by the Borwin software system.

#### 2.2.2. Fatty Acid Composition

The fatty acid composition was determined as methyl esters (FAME) according to O’Fallon et al. (2007) [35] on the finely ground cake (400 mg) and diets (800 mg), using nonadecanoic methyl ester as the internal standard. FAMEs were injected into a gas chromatograph Agilent 7890 GC system (Palo Alto, CA) equipped with an on-column injector, and an FID (Flame Ionization Detector) detector. The separation was performed on a 100% dimethylpolysiloxane column (CP-Sil88 for FAME, 100 m × 0.25 mm × 25 μm) adopting the conditions described in Placha et al. (2019) [36]. FAMEs were identified by comparison of their retention times with known standards (37-component FAME mix, Supelco 47885-U) and expressed as g/kg of cake or diet.

#### 2.2.3. Antinutritional Compounds

Glucosinolates (GSLs) were extracted and assayed from camelina cake and diets as reported in Russo and Reggiani (2017) [37]. The determination of sinapine was performed according to the method described by Bjerg et al. (1984) [38] and separated by HPLC as described by Clausen et al. (1985) [39] and modified by Russo and Reggiani (2017) [37].

#### 2.2.4. Volatile Organic Compounds (VOCs)

To detect the presence of volatile sulfur compounds from camelina cake and compounds derived from lipid oxidation the Solid Phase Micro Extraction Gas Chromatography-Mass Spectrometry (SPME-GC-MS) analysis was carried out. The analysis was performed on 5 g of cake or diet by means of a Combi-Pal automated sampler (CTC Analytics, Zwingen, Switzerland) equipped with DVB/CAR/PDMS 50/30 μm fiber (Supelco, Bellefonte, PA, USA) and coupled to a GC-MS (6890N/5973N Agilent Technologies, Inc., Wilmington, DE, USA) adopting the conditions described in Battelli et al. (2019) [40]. Data are expressed as arbitrary units of the area of the quant ion of each compound.

### 2.3. Data Collection

#### 2.3.1. Birds Performance and Welfare

Hens were monitored daily. The number of eggs was recorded daily; a sample of 10 eggs per pen were weighed weekly, as well as mortality and feed consumption were recorded. The welfare was assessed according to the Welfare Quality^®^ Assessment protocol for poultry [41]. At 23, 39, and 51 weeks of age, 10 layers per pen were randomly selected, weighted, and evaluated for the status of feathers in five body areas (head-neck, back, tail, wings, vent-cloaca). Body areas were scored for the severity of feather conditions from 1 (very poor condition) to 4 (very good condition) and wounds from 1 (presence) to 2 (absence). Keel bone deviations and fractures, as well as keel bone tips deviation were scored. Footpad lesions and hyperkeratosis were scored from 1 (presence) to 2 (absence), and incidence of wounds and missing toes were recorded. Comb color was scored from 1 (pale) to 2 (red).

#### 2.3.2. Eggshell Quality

Eggshell quality was evaluated at two different moments of laying period, at week 24 (before reaching the laying peak), and at week 43 (during the plateau). At each sampling time 20 eggs/treatment with a shape index (breadth/length × 100) of 78.5–79.5 have been weighed and submitted to a destructive mechanical test. Eggs were placed vertically on an egg holder under compression plate. Rupture force and deformation at rupture were measured by quasistatic compression at a constant compression speed of 20 mm/min using a dynamometer (Mod. Z005 Zwick Roell, Ulm, Germany) fitted with a 100 N load and a 30 mm Ø flat compression plate and supported by TestXpert (v 11.02) testing software (Zwick Roell, Ulm, Germany). Specific deformation and rupture energy were calculated as in Nedomova et al. 2009 [42]. After compression test, eggshells were rinsed, air-dried, and weighed. Egg surface, calculated as in Narushin (2005) [43], was used to estimate shell thickness as in Mabe et al. (2003) [27].

#### 2.3.3. Statistical Analysis

The data on performance and welfare were analyzed by one-way ANOVA with three treatments using SPSS (2017) [44]. Significant differences among treatment means were separated using LSD test at *p* < 0.05. The effect of diet and hens age on shell resistance was evaluated with three levels (0, 10, 20%) of camelina cake supplements and two levels (24 and 43 weeks) of hen age. Data were subjected to 3 × 2 ANOVA using General Linear Model (GLM) procedure [44]. In the case of significant interaction (*p* < 0.05), main effects of factors were not discussed and grouping of means was performed using Tukey’s test (95%).

### 2.4. Ethical Statement

The protocol received approval by the Ethical Committee of the University of Milan (approval number OPBA_11_2016). This study did not induce disease, injury, pain, or distress in laying hens.

## 3. Results and Discussion

### 3.1. Characterization of Camelina Cake and Diets

The result of the analysis of camelina cake, used in this study, is presented in Table 1. The camelina cake composition is comparable with those reported in earlier experiments [18,19,21]. Our analyses reconfirmed the high protein content in camelina cake (37.17%) and also the known specific profile of FA in the residual camelina oil (rich in n-3 polyunsaturated fatty acids (PUFA)), which makes camelina cake a potential source of protein and healthy polyunsaturated FA for poultry. Regarding the experimental diets, they were formulated to provide the metabolizable energy as the control diet (Table 2).

#### 3.1.1. Antioxidants

The antioxidant activity calculated by DPPH scavenging activity in cake and diets is shown in Table 1 and Table 3. The camelina cake had a very high antioxidant activity that is due to the high content of phenolic substances. It is interesting to note that the antioxidant activity of the control diet is relatively low and the addition of camelina in the diets increases the capacity of scavenging (Table 3). This is associated with an increase in phenolic compounds (TPC, flavonoids, and tannins) in diets containing camelina. To this, it must be added the contribution of tocopherols present in the camelina cake oil, which enhance the antioxidant capacity of the diets. In fact, these compounds resulted higher in CAM10 and CAM20 than in the control diet (Table 3).

#### 3.1.2. Fatty Acid Composition

The fatty acid content of camelina cake and diets is shown in Table 1 and Table 3. Regarding the three different diets, the effect of the increasing camelina cake is remarkable already in the CAM10 diet. The corresponding ratio n-6/n-3 decreases from 15.8 (C) to 2.53 and 1.57 in CAM10 and CAM20 respectively. Consequently, an important improvement in the nutritional quality of the eggs was achieved (Battelli et al., in preparation).

#### 3.1.3. Antinutritional Compounds

Glucosinolates and sinapine are the main anti-nutritive compounds in camelina [12,37] and may have negative effects on palatability, feed consumption, growth, and egg production [19,20,45]. The cake of the camelina line used in this work resulted to contain 15.5 mmol/kg DW of total GSLs (Table 1) which is far below the mean camelina content (30 mmol/kg DW) reported by Russo and Reggiani, (2017) [37]. In control feed (C), GSLs were absent while in camelina diets (CAM10 and CAM20) their content was 1.66 and 3.37 mmol/kg DW, respectively (Table 3), which is below the suggested limit reported for hens diet supplemented with rapeseed (4 mmol/kg [46]). The content of the other antinutritional metabolite sinapine was evaluated on cake and diets (Table 1 and Table 3). In the camelina cake, it was 2.26 g/kg, which is quite low when compared with other *Brassicaceae* plants, such as rapeseed, with a sinapine content of 7 g/kg [47]. Sinapine was negligible in the control feed (C) and its amount was very low in the CAM10 (0.12 g/kg) and CAM20 (0.46 g/kg) diets.

#### 3.1.4. Volatile Organic Compounds

**Figure 1:**
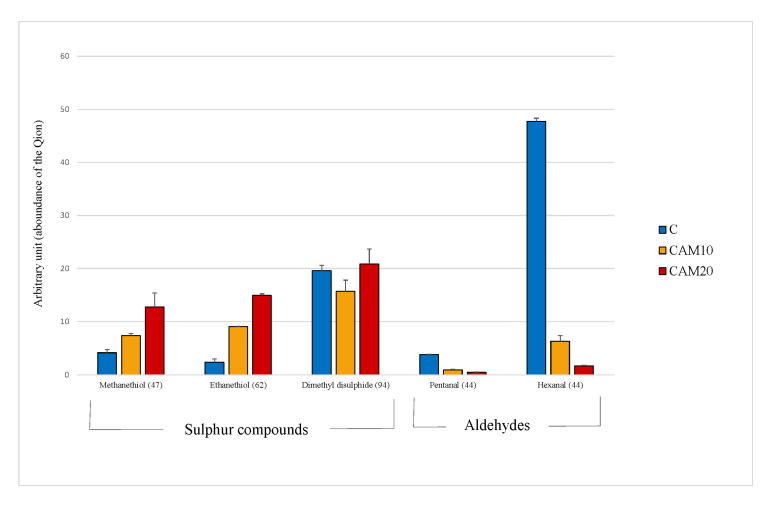
Volatile compounds recognized in the Control and camelina diets (CAM10 and CAM20). Data expressed as arbitrary units refer to the peak area of the Quant ion (in brackets).

The principal volatile compounds detected in camelina diets were those derived from the oxidation of the polyunsaturated fatty acids (PUFA), such as hexanal, and the volatile sulfur compounds contained in *Brassicaceae* family, such as methanethiol and ethanethiol [48]. It must be stressed that the data obtained by VOCs analysis are expressed in arbitrary units, thus the results were evaluated only relatively among diets. In Figure 1 is clearly shown how sulfur compound levels increase with the percentage of camelina in diet, especially for methanethiol (×1.8 and ×3.0 fold, respectively). On the other hand, the antioxidant compounds contained in camelina cake (Table 1) dramatically decrease the oxidation products, enhancing the nutritional value of the diet. The level of hexanal, in fact is strongly reduced in CAM10 and CAM20 diets (−87% and −96%, respectively), even though the PUFA highly susceptible of oxidation, increased in the two diets (Table 3).

### 3.2. Birds Performance and Welfare

The number of repetition (two pens for treatment) was decided in order to avoid any change in management and environment of the animals that could bias the results. Studies with many repetitions with few animals (often kept in cages) x repetition generally aim to test the effect of some new ingredients in the diet. As the positive effect of camelina was already proven by several studies [18,19,21,22,49], the novelty of our paper is that camelina cake used conditions close to the farm ones, for a long period (31 weeks) and with a high percentage (up to 20%) of camelina cake in the diet. In light of this consideration, it is worth noticing that papers addressing applied research questions use the same approach [50].

The number of eggs was not affected by the percentage of camelina cake in the diet (Table 4). Feed consumption was 128.57, 122.35, 114.04 g/hen per day for C, CAM10, and CAM20 hens, respectively. These data were similar to the results of Cherian et al. (2009) [21] that used camelina cake in three treatments (5%, 10%, and 15%); authors found that hen-day egg production was lowest for CAM15 and no differences among the other treatments. Feed consumption was 106.5, 105.2, 97.7, and 98.7 g/hen per day for control, CAM5, CAM10, and CAM15 birds, respectively. Kakani et al. (2012) [49] examined the effects of feeding extruded defatted camelina cake (5% and 10%) to layers and they found no differences in percent hen-day egg production and feed consumed. Frame et al. (2007) [51] reported a reduction in feed intake when the diets contained more than 5% camelina in the diets of young turkeys. In this study, the use of camelina cake up to 20% in the diet did not negatively affect the production, feed consumption, and feed conversion rate (Table 4), suggesting that camelina cake could be an economic sustainable source of protein for poultry.

**Figure 2:**
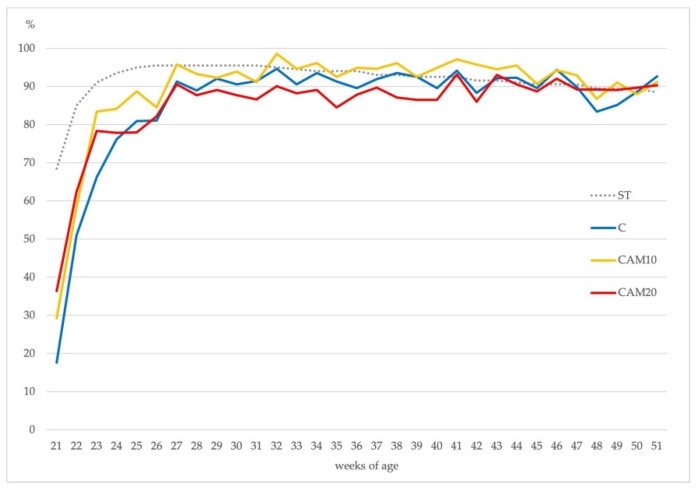
Hen-day egg production (%) in the Control diet (C) and camelina diets (CAM10 and CAM20) vs. Hy-Line^®^ brown standard (ST).

Eggs production rate (eggs laid × 100/number of hens) was similar among the three treatments according to the Hy-Line^®^ standard. As shown in Figure 2, all treatments peak in egg production at the same time (26–27 weeks of age), the persistence of the deposition until the end of the production cycle was characterized by slowly reducing egg production; this picture is common in most layer flocks [52].

**Figure 3:**
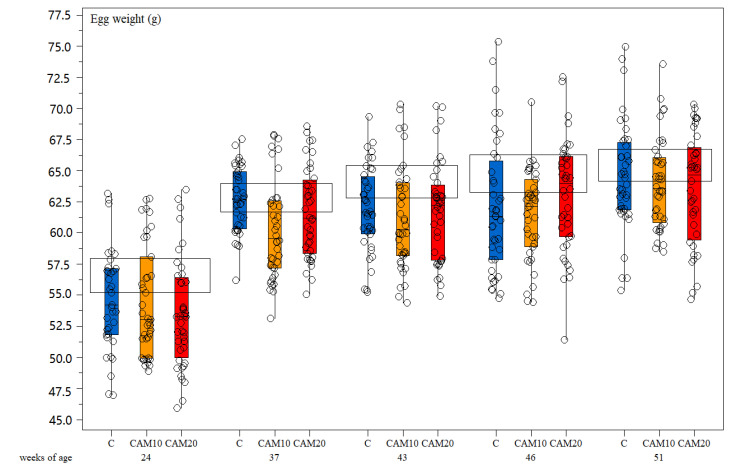
Box-and-whisker plots including individual measurements of egg weight (g). Effect of hen age (weeks) and different camelina cake level in the diet (Control diet—C, camelina diets—CAM10 and CAM20). The horizontal boxes refer to the medium values for Hy-Line^®^ brown standard.

Egg weight did not show any significant difference among treatments—neither to Hy-Line^®^ standard during the rearing period (Figure 3). Laying hens mortality was not observed in any of the treatment groups during the experimental period. Regarding welfare, the plumage condition was generally good for all treatments during the whole period. The slight reduction in the score seems to be more related to the age than to the diets [53]. Wounds were almost absent; plumage and footpad conditions were good, and the growth performance of the birds was similar to the control (Table 5).

### 3.3. Eggshell Mechanical and Quality Traits

Altuntaş and Şekeroğlu (2008) [54] demonstrated that rupture force depended highly on egg shape, whereas other authors [42,55] did not find any relation between mechanical behavior and egg geometry. However, to avoid any possible influence of egg shape on results, all eggs submitted in our study to compression test had a shape index in the range of 78.5–79.5.

Results of two factor (3 × 2) ANOVA related to quasistatic compression test are shown in Table 6. No significant interaction was detected between hen age and diet (*p*_HxD_ > 0.05) on shell percentage and thickness. Results indicated a significant main effect for hen age on shell percentage (*p*_H_ < 0.001) and for diet on shell thickness (*p*_D_ < 0.05). Shell percentage decreased in all groups with progressing age while shell thickness had significantly higher overall values for CAM10 compared to CAM20 group. All response variables of compression test show, instead, a significant interaction of factors (*p*_HxD_ < 0.05). The effect of the level of hen age is not the same across the levels of camelina cake in the diet. Results for rupture force, deformation, and rupture energy for control group C show a decreasing trend with a significant difference of all variables at week 43 compared to week 24. Eggs from CAM10 and CAM20 groups instead show a more stable performance.

As hens got older, shell percentage decreased subsequently in all groups, following the well-known occurrence of a faster growing rate for egg weight compared to shell weight as age advances. Without a simultaneous improvement in carbonate deposition in the shell, the non-proportional rate of growth and the resulting decreased shell percentage turn out in a lower resistance to fracture of eggs from older hens [25,31,56]. These results, together with the uneven effect of the level of hen age across the levels of the diet on compression test results, suggest to us a protective role of camelina cake for eggshells of older hens.

Different studies on camelina cake supplementation in the range of 3–15% [21,57,58] demonstrated little effect on shell thickness and percentage. Ten percent of full fat camelina in the diet [59] likewise did not affect these shell traits. Kakani et al. (2012) [49] reported a significant difference in shell thickness between the two camelina groups; camelina 10% showed significantly lower shell thickness than camelina 5%.

Moreover, the presence of camelina cake at 5 and 10% significantly increased the shell resistance to fracture of both camelina groups. Our results are in line with this last author, taking into account, however, the differences of the two studies in terms of hen genotype, age, and camelina percentage in the diet. Bioactive molecules (phenols, tocopherols, and flavonoids) contained in camelina cake [60] could be involved in protecting eggshell as hens become older. Synthetic antioxidants of vitamin E [61] and flavonoids supplements [62,63] already demonstrated, in fact, a positive effect on eggshell of heat-stressed or aged hens. Research should, therefore, be carried out and widened to understand better how amino acids, minerals, micronutrients, and bioactive molecules contained in camelina cake relate to the organic components of true shell and shell membrane, to shell composition, to calcium metabolism and availability, as well as to shell ultrastructure.

## 4. Conclusions

Feeding trials conducted in this work showed that inclusion in the diet of up to 20% of camelina cake containing 15.5 mmol of GSLs/kg did not have any negative impact—neither on the performance nor on the welfare—of laying hens. Moreover, the mechanical eggshell quality traits of CAM10 and CAM20 groups had a more stable performance over time. It should be underlined that the hens were reared close to farm conditions and received a diet containing camelina cake for a longer period (31 weeks), in comparison to the feeding period reported in other papers. Thus, camelina cake might be a suitable and sustainable alternative source of protein, antioxidant compounds, and PUFA in laying hens, with a positive economic impact.

## Figures and Tables

**Table 1:** Characterization of camelina cake.

Parameter	Amount	Parameter	Amount
Gross Energy, MJ/kg	17.88	**Antioxidant activity and compounds**	
Proximate analysis *		DPPH radical scavenging activity, %	76.5
Crude protein, %	37.17	Total phenolic content (TPC), mgCAE **/g	22.7
Crude fat, %	19.17	Flavonoids, g/kg	4.83
Crude fiber, %	10.72	Tannins, g/kg	6.40
Ash, %	6.80	Tocopherols, mg/kg	687
Neutral-detergent fiber (NDF), %	35.63	**Antinutritional compounds**	
**Fatty acid composition**		Glucosinolates, mmol/kg	15.5
saturated fatty acids (SFA), g/kg	16.1	Sinapine, g/kg	2.26
monounsaturated fatty acids (MUFA), g/kg	44.4		
n-6 polyunsaturated fatty acids (PUFA), g/kg	34.1		
n-3 PUFA, g/kg	63.6		
n-6/n-3	0.54		

* Proximate analyses were performed at the Associazione Regionale Allevatori della Lombardia (ARAL) Laboratory (Crema, Italy). ** Caffeic Acid Equivalents

**Table 2:** Composition of experimental diets and calculated nutrient contents expressed as fed.

	Control (C)	Camelina 10% (CAM10)	Camelina 20% (CAM20)
Ingredients, g/kg			
Corn	537.5	524.0	508.5
Soybean meal	320	247	174
Calcium carbonate	95	95	95
Camelina cake	0	100	200
Soybean oil	25	11.5	0
Dicalcium phosphate	12	12	12
Vitamin premix *	5	5	5
Salt	4	4	4
Methionine	1.5	1.5	1.5
Metabolizable Energy, MJ/kg			
	11.05	10.88	10.77
Analytic Composition %			
Crude Protein	18.08	18.32	18.54
Crude Fat	5.30	5.51	5.90
Crude Fiber	3.17	3.69	4.20
Ash	14.12	14.26	14.40
L-Lysine	0.90	0.96	1.02
Methionine	0.43	0.45	0.46
Methionine + Cysteine	0.75	0.66	0.56
L-Threonine	0.71	0.75	0.79
Ca	4.15	4.15	4.16
P	0.56	0.57	0.58
Available P	0.35	0.33	0.32
Na	0.17	0.16	0.16
Cl	0.22	0.22	0.22

* Vitamins: A 10.000 IU; E 30.00 mg; D3 3000 IU; K 2.50 mg; B1 2.00 mg; B2 6.00 mg; B6 4.00 mg; B12 0.02 mg; H 0.05 mg; PP 30.00 mg; Folic Acid 1.00 mg; D-Pant 8.00 mg; Coline 600.00 mg Fe 40.00 mg; Cu 12.00; Zn 75.00 mg; Mg 150.00 mg; Se 0.30 mg; I 1.20 mg.

**Table 3:** Antioxidant capacity (DPPH), total phenolic compounds (TPC), flavonoids, tannins, tocopherol content, fatty acid composition, and antinutritional compounds (mean value ± SE) of the experimental diets. Control = corn-soybean meal basal diet; CAM10 = basal diet containing camelina cake at 10%; CAM20 = basal diet containing camelina cake at 20%.

Experimental Diets			
Parameter	Control (C)	CAM10	CAM20
DPPH, %	28.2 ± 0.30	70.2 ± 0.20	74.6 ± 0.10
TPC, mgCAE/g	8.5 ± 0.10	10.9 ± 0.10	12.3 ± 0.20
Flavonoids, g/kg	2.24 ± 0.03	4.44 ± 0.06	7.92 ± 0.10
Tannins, g/kg	3.38 ± 0.05	4.18 ± 0.04	4.52 ± 0.05
Tocopherols, mg/kg	321 ± 20	464 ± 12	537 ± 10
SFA, g/kg	5.6 ± 0.20	7.3 ± 0.05	8.0 ± 0.08
MUFA, g/kg	7.5 ± 0.26	11.6 ± 0.00	12.0 ± 0.04
n-6 PUFA, g/kg	15.2 ± 0.62	21.9 ± 0.27	22.5 ± 0.05
n-3 PUFA, g/kg	1.0 ± 0.04	8.7 ± 0.22	14.3 ± 0.02
n-6/n-3	15.83	2.53	1.57
Glucosinolates, mmol/kg	0	1.66 ± 0.08	3.37 ± 0.02
Sinapine, g/kg	0	0.12 ± 0.01	0.46 ± 0.01

**Table 4:** Effect of camelina cake in laying hen diets on average daily intake, egg production, egg weight, and feed conversion ratio (FCR) from 21 to 51 weeks of age (Control diet—C, camelina diets—CAM10 and CAM20; mean ± SE).

Performance	C	CAM10	CAM20
Average Daily Intake (g/day/hen)	128.57 ± 4.94	122.35 ± 5.03	114.04 ± 6.12
Egg production/week	464.16 ± 14.70	499.00 ± 13.40	473.94 ± 11.00
Egg weight (g)	62.64 ± 4.26	62.16 ± 3.77	61.79 ± 3.72
FCR	2.44 ± 0.10	2.19 ± 0.12	2.22 ± 0.11
Initial hen’s weight (g)	1872.5 ± 30.9	1765.0 ± 34.6	1782.5 ± 35.4
Final hen’s weight (g)	2060.0 ± 40.5	2065.0 ± 35.2	2000.0±46.7

**Table 5:** Plumage condition in the five areas considered using Welfare Quality^®^ scoring system. Control diet (C) and camelina diets (CAM10 and CAM20).

Body Part	Week	C	CAM10	CAM20
Head-Neck	23	4.0	4.0	4.0
	39	4.0	4.0	3.6
	51	3.7	3.6	3.9
Back	23	4.0	4.0	4.0
	39	4.0	4.0	3.6
	51	3.7	3.6	3.9
Tail	23	4.0	4.0	4.0
	39	4.0	4.0	4.0
	51	4.0	3.9	4.0
Wings	23	4.0	4.0	4.0
	39	4.0	4.0	4.0
	51	4.0	3.6	4.0
Vent-cloaca	23	4.0	4.0	4.0
	39	4.0	4.0	4.0
	51	4.0	4.0	4.0

**Table 6:** Effect of hen age and different camelina cake level in the diet on compression test response variables (Rupture Force, Specific deformation, and Rupture Energy), and shell traits (shell percentage and thickness) of eggs with shape index 78.5–79.5. Control diet (C) and camelina diets (CAM10 and CAM20).

Factors		Response Variables									
D ^1^	H ^2^ (w)	Fmax ^3^ (N)		% Def ^4^		Ea ^5^ (N × mm)		% Shell ^6^		T ^7^ (mm)	
C		47.6		0.65		9		10.1		0.37	^a,b^
CAM10		48.4		0.68		9		10.2		0.38	^a^
CAM20		47.6		0.68		9		9.9		0.36	^b^
	24	50.5		0.74		10		10.3	^a^	0.37	
	43	45.2		0.60		8		9.8	^b^	0.37	
C	24	53.2	^a^	0.75	^a^	11	^a^	10.5		0.38	
	43	42.3	^b^	0.55	^c^	7	^c^	9.7		0.36	
CAM10	24	50.6	^a^	0.73	^a,b^	10	^a,b^	10.4		0.37	
	43	46.3	^a,b^	0.62	^b,c^	8	^b,c^	10.0		0.38	
CAM20	24	48.2	^a,b^	0.73	^a,b^	9	^a,b^	10.1		0.36	
	43	46.9	^a,b^	0.62	^b,c^	8	^b,c^	9.7		0.37	
Pooled SEM		2.235		0.006		0.252		0.222		0.0003	
ANOVA	***p***										
H		<0.001		<0.001		<0.001		<0.001		0.931	
D		0.875		0.408		0.845		0.118		0.043	
H × D		0.033		0.048		0.026		0.320		0.093	

^a–c^ Statistically significant differences in columns (*p* ≤ 0.05) are indicated by different superscripts; ^1^ D = diet; ^2^ H = Hen age expressed in weeks; ^3^ Fmax = measured Rupture Force; ^4^ %Def = Specific Deformation = 100 × measured Deformation at rupture / egg length; ^5^ Ea = Rupture Energy = Fmax × measured Deformation at rupture / 2; ^6^ % shell = 100 × eggshell weight / total egg weight; ^7^ T = Shell Thickness = 10 × eggshell weight /(Surface x 2.3) where Surface is calculated as in Narushin 2005 [43].

## References

[1] Parliament T.E., Parliament E., Community E.E. (2018). European Parliament 2014–2019.

[2] Smith N.O. (1997). Crops for Industry and Energy in Europe.

[3] Gugel R.K., Falk K.C. (2006). Agronomic and seed quality evaluation of Camelina sativa in western Canada. Can. J. Plant Sci..

[4] Berti M., Gesch R., Eynck C., Anderson J., Cermak S. (2016). Camelina uses, genetics, genomics, production, and management. Ind. Crops Prod..

[5] Zohary D., Hopf M. (2000). Domestication of Plants in the Old World: The Origin and Spread of cultivated Plants in West Asia, Europe and the Nile Valley.

[6] Vollmann J., Moritz T., Kargl C., Baumgartner S., Wagentristl H. (2007). Agronomic evaluation of camelina genotypes selected for seed quality characteristics. Ind. Crops Prod..

[7] Masella P., Martinelli T., Galasso I. (2014). Agronomic evaluation and phenotypic plasticity of Camelina sativa growing in Lombardia, Italy. Crop Pasture Sci..

[8] Zanetti F., Eynck C., Christou M., Krzyżaniak M., Righini D., Alexopoulou E., Stolarski M.J., Van Loo E.N., Puttick D., Monti A. (2017). Agronomic performance and seed quality attributes of Camelina (*Camelina sativa* L. crantz) in multi-environment trials across Europe and Canada. Ind. Crops Prod..

[9] Fröhlich A., Rice B. (2005). Evaluation of Camelina sativa oil as a feedstock for biodiesel production. Ind. Crops Prod..

[10] Zaleckas E., Makarevičienė V., Sendžikienė E. (2012). Possibilities of using Camelina sativa oil for producing biodiesel fuel. Transport.

[11] Agusdinata D.B., Zhao F., Ileleji K., DeLaurentis D. (2011). Life cycle assessment of potential biojet fuel production in the United States. Environ. Sci. Technol..

[12] Matthäus B., Zubr J. (2000). Variability of specific components in Camelina sativa oilseed cakes. Ind. Crops Prod..

[13] Ibrahim F.M., El Habbasha S.F. (2015). Chemical composition, medicinal impacts and cultivation of camelina (*Camelina sativa*). Int. J. PharmTech. Res..

[14] Cherian G. (2012). Camelina sativa in poultry diets: Opportunities and challenges. Biofuel co-Products as Livest. Feed Oppor. Challenges.

[15] Colombini S., Broderick G.A., Galasso I., Martinelli T., Rapetti L., Russo R., Reggiani R. (2014). Evaluation of Camelina sativa (L.) Crantz meal as an alternative protein source in ruminant rations. J. Sci. Food Agric..

[16] Zubr J., Matthäus B. (2002). Effects of growth conditions on fatty acids and tocopherols in Camelina sativa oil. Ind. Crops Prod..

[17] Abramovič H., Butinar B., Nikolič V. (2007). Changes occurring in phenolic content, tocopherol composition and oxidative stability of Camelina sativa oil during storage. Food Chem..

[18] Aziza A.E., Quezada N., Cherian G. (2010). Feeding Camelina sativa meal to meat-type chickens: Effect on production performance and tissue fatty acid composition. J. Appl. Poult. Res..

[19] Ryhänen E., Perttilä S., Tupasela T., Valaja J., Eriksson C., Larkka K. (2007). Effect of Camelina sativa expeller cake on performance and meat quality of broilers. J. Sci. Food Agric..

[20] Pekel A.Y., Patterson P.H., Hulet R.M., Acar N., Cravener T.L., Dowler D.B., Hunter J.M. (2009). Dietary camelina meal versus flaxseed with and without supplemental copper for broiler chickens: Live performance and processing yield. Poult. Sci..

[21] Cherian G., Campbell A., Parker T. (2009). Egg quality and lipid composition of eggs from hens fed Camelina sativa. J. Appl. Poult. Res..

[22] Quentin M., Bouvarel I., Picard M. (2005). Effects of crude protein and lysine contents of the diet on growth and body composition of slow-growing commercial broilers from 42 to 77 days of age. Anim. Res..

[23] Hetland H., Choct M., Svihus B. (2004). Role of insoluble non-starch polysaccharides in poultry nutrition. Worlds Poult. Sci. J..

[24] Tarlton J.F., Wilkins L.J., Toscano M.J., Avery N.C., Knott L. (2013). Reduced bone breakage and increased bone strength in free range laying hens fed omega-3 polyunsaturated fatty acid supplemented diets. Bone.

[25] Roberts J.R. (2004). Factors affecting egg internal quality and egg shell quality in laying hens. J. Poult. Sci..

[26] Ketta M., Tůmová E. (2016). Eggshell structure, measurements, and quality-affecting factors in laying hens: A review. Czech J. Anim. Sci..

[27] Mabe I., Rapp C., Bain M.M., Nys Y. (2003). Supplementation of a corn-soybean meal diet with manganese, copper, and zinc from organic or inorganic sources improves eggshell quality in aged laying hens. Poult. Sci..

[28] Herkeľ R., Gálik B., Bíro D., Rolinec M., Šimko M., Juráček M., Arpášová H., Hanušovský O. (2017). The effect of essential oils on quality and mineral composition of eggshell. Acta Fytotech. Zootech..

[29] Pavlović Z., Miletić I., Jokić Ž., Pavlovski Z., Škrbić Z., Šobajić S. (2010). The effect of level and source of dietary selenium supplementation on eggshell quality. Biol. Trace Elem. Res..

[30] Świątkiewicz S., Arczewska-Włosek A., Krawczyk J., Puchała M., Jozefiak D. (2015). Dietary factors improving eggshell quality: An updated review with special emphasis on microelements and feed additives. Worlds Poult. Sci. J..

[31] Rayan G.N., Galal A., Fathi M.M., El-Attar A.H. (2010). Impact of layer breeder flock age and strain on mechanical and ultrastructural properties of eggshell in chicken. Int. J. Poult. Sci..

[32] Brand-Williams W., Cuvelier M.-E., Berset C. (1995). Use of a free radical method to evaluate antioxidant activity. LWT-Food Sci. Technol..

[33] Russo R., Reggiani R. (2018). Antioxidants in flour of the oilseed crop *Camelina sativa* (L.) Crantz. Int. J. Plant Biol..

[34] Gimeno E., Calero E., Castellote A.I., Lamuela-Raventos R.M., De la Torre M.C., Lopez-Sabater M.C. (2000). Simultaneous determination of α-tocopherol and β-carotene in olive oil by reversed-phase high-performance liquid chromatography. J. Chromatogr. A.

[35] O’fallon J.V., Busboom J.R., Nelson M.L., Gaskins C.T. (2007). A direct method for fatty acid methyl ester synthesis: Application to wet meat tissues, oils, and feedstuffs. J. Anim. Sci..

[36] Placha I., Ocelova V., Chizzola R., Battelli G., Gai F., Bacova K., Faix S. (2019). Effect of thymol on the broiler chicken antioxidative defence system after sustained dietary thyme oil application. Br. Poult. Sci..

[37] Russo R., Reggiani R. (2017). Glucosinolates and Sinapine in camelina meal. Proc. Food Nutr. Sci..

[38] Bjerg B., Olsen O., Rasmussen K.W., Srensen H. (1984). New principles of ion-exchange techniques suitable to sample preparation and group separation of natural products prior to liquid chromatography. J. Liq. Chromatogr..

[39] Clausen S., Larsen L.M., Plöger A., Soerensen H. (1985). Aromatic choline esters in rapeseed. World Crop. Prod. Util. Descr..

[40] Battelli G., Scano P., Albano C., Cagliani L.R., Brasca M., Consonni R. (2019). Modifications of the volatile and nonvolatile metabolome of goat cheese due to adjunct of non-starter lactic acid bacteria. LWT.

[41] Quality W. (2009). Welfare Quality^®^ Assessment Protocol for Cattle.

[42] Nedomova S., Severa L., Buchar J. (2009). Influence of hen egg shape on eggshell compressive strength. Int. Agrophys..

[43] Narushin V.G. (2005). Egg geometry calculation using the measurements of length and breadth. Poult. Sci..

[44] Corportation I.B.M. (2017). IBM SPSS Statistics for Windows (Version 25.0 Armonk).

[45] Acamovic T., Gilbert C., Lamb K., Walker K.C. (1999). NUTRITION Nutritive value of Camelina sativa meal for poultry. Br. Poult. Sci..

[46] Tripathi M.K., Mishra A.S. (2007). Glucosinolates in animal nutrition: A review. Anim. Feed Sci. Technol..

[47] Matthäs B. (1997). Antinutritive compounds in different oilseeds. Lipid/Fett.

[48] Chin H.-W., Lindsay R.C. (1994). Mechanisms of formation of volatile sulfur compounds following the action of cysteine sulfoxide lyases. J. Agric. Food Chem..

[49] Kakani R., Fowler J., Haq A.-U., Murphy E.J., Rosenberger T.A., Berhow M., Bailey C.A. (2012). Camelina meal increases egg n-3 fatty acid content without altering quality or production in laying hens. Lipids.

[50] Geng A.L., Liu H.G., Zhang Y., Zhang J., Wang H.H., Chu Q., Yan Z.X. (2020). Effects of indoor stocking density on performance, egg quality, and welfare status of a native chicken during 22 to 38 weeks. Poult. Sci..

[51] Frame D.D., Palmer M., Peterson B. (2007). Use of Camelina sativa in the diets of young turkeys. J. Appl. Poult. Res..

[52] Pescatore T., Jacob J. (2012). Kentucky 4-H Poultry: Evaluating Egg-Laying Hens. http://www2.ca.uky.edu/agcomm/pubs/4aj/4aj07po/4aj07po.PDF.

[53] Iqbal Z., Drake K., Swick R.A., Taylor P.S., Perez-Maldonado R.A., Ruhnke I. (2020). Effect of pecking stones and age on feather cover, hen mortality, and performance in free-range laying hens. Poult. Sci..

[54] Altuntaş E., Şekeroğlu A. (2008). Effect of egg shape index on mechanical properties of chicken eggs. J. Food Eng..

[55] Anderson K.E., Tharrington J.B., Curtis P.A., Jones F.T. (2004). Shell characteristics of eggs from historic strains of single comb white leghorn chickens and the relationship of egg shape to shell strength. Int. J. Poult. Sci..

[56] Sirri F., Zampiga M., Berardinelli A., Meluzzi A. (2018). Variability and interaction of some egg physical and eggshell quality attributes during the entire laying hen cycle. Poult. Sci..

[57] Vasilachi A., Criste R.D., Cornescu M.G., Olteanu M., Panaite T.D., Sredanović S.A., Spasevski N. Effect of the dietary camelina meal on layer performance. Proceedings of the 6th Central European Congress on Food-CEFood Congress; Institute of Food Technology.

[58] Aziza A.E., Panda A.K., Quezada N., Cherian G. (2013). Nutrient digestibility, egg quality, and fatty acid composition of brown laying hens fed camelina or flaxseed meal. J. Appl. Poult. Res..

[59] Cherian G., Quezada N. (2016). Egg quality, fatty acid composition and immunoglobulin Y content in eggs from laying hens fed full fat camelina or flax seed. J. Anim. Sci. Biotechnol..

[60] Quezada N., Cherian G. (2012). Lipid characterization and antioxidant status of the seeds and meals of Camelina sativa and flax. Eur. J. lipid Sci. Technol..

[61] Al-Harthi M.A. (2014). The effect of natural and synthetic antioxidants on performance, egg quality and blood constituents of laying hens grown under high ambient temperature. Ital. J. Anim. Sci..

[62] Ni Y., Zhu Q., Zhou Z., Grossmann R., Chen J., Zhao R. (2007). Effect of dietary daidzein on egg production, shell quality, and gene expression of ER-α, GH-R, and IGF-IR in shell glands of laying hens. J. Agric. Food Chem..

[63] Liu Y., Li Y., Liu H.-N., Suo Y.-L., Hu L.-L., Feng X.-A., Zhang L., Jin F. (2013). Effect of quercetin on performance and egg quality during the late laying period of hens. Br. Poult. Sci..

